# Efficient and Robust Method to Detect the Location of Macular Center Based on Optimal Temporal Determination

**DOI:** 10.3390/jimaging8120313

**Published:** 2022-11-22

**Authors:** Helmie Arif Wibawa, Agus Harjoko, Raden Sumiharto, Muhammad Bayu Sasongko

**Affiliations:** 1 Department of Informatics, Faculty of Science and Mathematics, Diponegoro University, Semarang 50275, Indonesia; 2 Department of Computer Science and Electronics, Faculty of Mathematics and Natural Sciences, Gadjah Mada University, Yogyakarta 55281, Indonesia; 3 Department of Ophthalmology, Faculty of Public Health Medicine and Nursing, Gadjah Mada University, Yogyakarta 55281, Indonesia

**Keywords:** macular center detection, temporal direction, macular ROI

## Abstract

The location of the macular central is very important for the examination of macular edema when using an automated screening system. The erratic character of the macular light intensity and the absence of a clear border make this anatomical structure difficult to detect. This paper presents a new method for detecting the macular center based on its geometrical location in the temporal direction of the optic disc. Also, a new method of determining the temporal direction using the vascular features visible on the optic disc is proposed. After detecting the optic disc, the temporal direction is determined by considering blood vessel positions. The macular center is detected using thresholding and simple morphology operations with optimum macular region of interest (ROI) direction. The results show that the proposed method has a low computation time of 0.34 s/image with 100% accuracy for the DRIVE dataset, while that of DiaretDB1 was 0.57 s/image with 98.87% accuracy.

## 1. Introduction

Diabetic macular edema (DME) often threatens the vision of people with diabetes when not treated properly, and fundus photos have been recommended for routine eye screening [1]. Analysis of fundus images with computer technology can make such examinations more effective and efficient.

**Figure 1:**
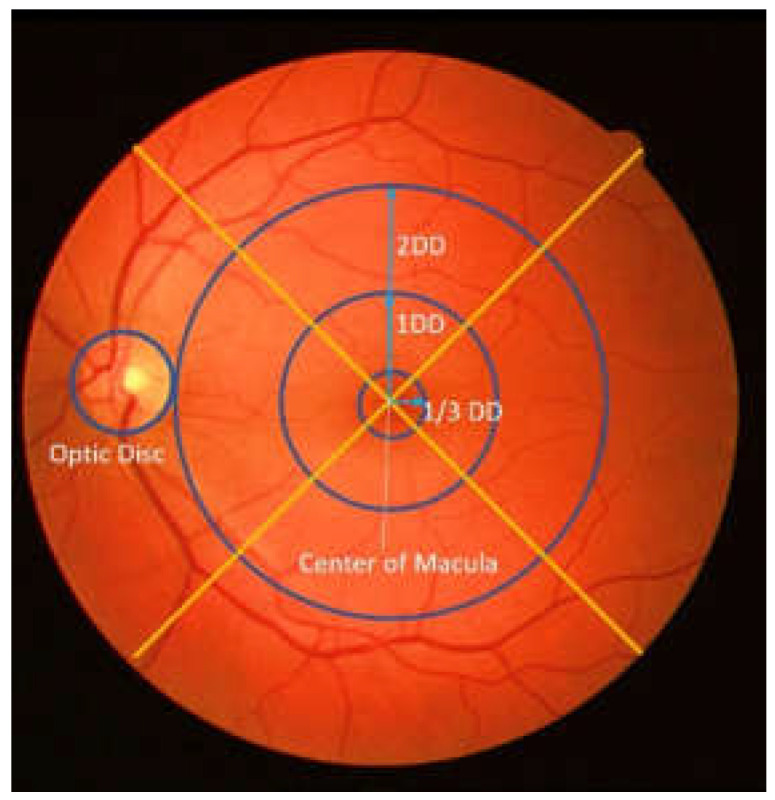
An example of polar coordinates in retinal image (DD: disc diameter).

DME severity is measured with polar coordinates that are determined by the appearance of a hard exudate on the retinal fundus image and its distance to the macular area [2,3,4]. The center of the macular is used as the focus in this polar coordinate system [5,6], which helps in measuring the distance of the hard exudate when it appears. Therefore, macular center detection is considered the initial step for their determination [7]. Figure 1 shows a polar coordinate system for the appearance and distribution of hard exudate in the macular area.

In the retinal fundus image, the macula is identified as the small circular portion with the darkest intensity [5] without the clear border of the optic disc area. Furthermore, as this area does not contain blood vessels [8], it is more difficult to detect the macular than anatomical structures such as the optic disc and blood vessels.

Therefore, the location of the macular center must be detected as the first step towards establishing the polar coordinates. In the past, the detection of the macular center has been performed using deep learning and traditional approaches. In one study, a deep-learning approach using Convolutional Neural Network (CNN) to simultaneously detect the optic disc and fovea [9] In another study, multi-stage segmentation of the fovea was performed with fully convolutional neural networks and explained that the fovea was segmented and localized using an end-to-end encoder–decoder network [10]. It has been found that deep-learning usage involves very complex parameters that influence each other, even though it provides high accuracy results [9]. Furthermore, the inspection and repair process in this approach is more complicated compared to the traditional method because of its black-box nature. This is consistent with [11,12,13,14], which stated that the deep-learning method requires large amounts of data to achieve optimal results. 

The traditional approach has been used to directly detect the macula based on the darkest intensity [15], while other studies have employed template matching to identify the fovea [16,17,18,19]. For example, a template based on the Gauss function was used in [16,19], while a histogram of the mean intensity was employed in [17]. A similar process was also utilized by [8] to perform matching for the extracted local features. Support Vector Machine (SVM) has also been used to determine macula candidates [3]. Furthermore, [20,21,22] employed geometric techniques in center detection and compared the location of the macular center to the optic disc or blood vessels. Zheng et al. [23] detected the macular center based on the location of the optic disc (OD) by using the temporal direction. However, the method failed to detect the macular center when the macular area was not clear.

It is important to note that the traditional methods mentioned above focus on determining and formulating specific characters for describing the fovea [24], such as the area with the darkest intensity or the fewest blood vessels. This formulation is difficult when conditions that do not fulfill the criteria are encountered, such as the appearance of hard exudate or a large black area in the fovea. The method proposed in this paper attempts to overcome these shortcomings and focuses on parameter formulation as well as the features for determining the macular center in the temporal area. Moreover, this method does not require large amounts of data to formulate an optimal model.

This paper also proposes an approach for obtaining the location of the macular center via determination of the optimum macular region of interest (ROI) based on the temporal direction. After the optic disc is detected, a feature for determining the temporal direction is extracted, which helps with accurate identification of the macular center location. The contributions of this study can be summarized as follows:

1. The macular center can be detected based only on its geometrical location in relation to the optic disc. This often leads to robust variations when detecting the intensity.
2. The method uses the inherent features in the optic disc to determine the temporal direction in which the macula is located, thereby making the process run faster.
3. Macular ROI with the right direction, location, and size reduces the detection area, facilitating a simpler detection process.

The study is presented in the following order: Section 2 describes the materials and methods used, including the sequence of processes for macular center detection. Section 3 and Section 4 respectively present the results and discussion, while the conclusions are contained in Section 5.

## 2. Materials and Methods

### 2.1. Materials

#### 2.1.1. Dataset

A total of four datasets were used in this study: 3 public datasets, namely DRIVE, DiaretDB1, and Messidor, and 1 local dataset. DRIVE [25] consists of 40 color images with 8-bit depth. The images have a 768 × 584 size with a 45° field of view (FOV).

DiaretDB1 contains 89 retinal images taken from Kuopio University Hospital. The images have a size of 1500 × 1552 pixels and were taken with a 50° FOV [26]. The Messidor dataset contains 1200 retinal images; of these, 212, 400, 588 images with a size of 2304 × 1536, 2240 × 1488, 1440 × 960 pixels, respectively, were included [27].

The images from the local dataset were taken from the Jogjakarta Eye Diabetic Study in the Community (JOGED.com) [28]. This local dataset has two sizes, namely 2124 × 2056 and 3696 × 2448, with 73 and 26 images, respectively.

#### 2.1.2. Environment

The experimental results were obtained using MATLAB R2018b on a computer with a 2.50 GHz Intel (R) Core (TM) i5– 4200 CPU, 4GB RAM, and Intel (R) HD Graphics 4600 graphics card.

### 2.2. Methods

**Figure 2:**
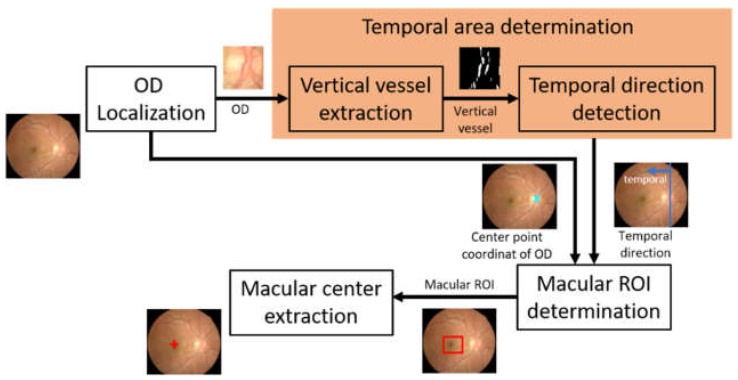
The flow of macular center point detection (OD: optic disc, ROI: region of interest).

In this study, the macular center was determined based on its geometrical location relative to the optic disc. The macula was considered as the area with the darkest intensity in the retinal image, and its center was located at 2.5 optic disc diameter (DD) temporally from the optic disc [23]. The search for the optic disc location and the temporal direction are prerequisites for determining the macula position. The method used for identifying the macular center consists of several main steps, which include optic disc localization, determining temporal area direction, identifying the macular region of interest (ROI), and macular center point coordinates extraction. Figure 2 shows the flow of macular center point detection.

#### 2.2.1. Optic Disc Localization

The OD center served as a reference point when determining the location of the macular coordinates. Furthermore, the blood vessels visible on the OD were useful for determining the direction of the temporal area on the retinal image [23]. It is important to note that the OD is an anatomical structure in the retinal image that has a higher light intensity compared to others, and this is the reason for it being localized through its intensity character. In this study, localization was conducted by combining the thresholding and morphological operation methods [29]. This thresholding technique was employed to determine the intensity value limit that distinguishes the OD from other areas. Meanwhile, the morphological operation was conducted to improve the thresholding results in order to provide an optimal OD area.

Before the OD localization process, image preprocessing was conducted, consisting of image resizing and intensity normalization. This image resizing was performed to equalize the image height between datasets for more uniformity and to reduce the processing time. The height of the image was resized to 565 pixels, while the width of the image was adjusted to the proportion of the input image. The value of 565 was taken from the height of the image in the DRIVE dataset, which has the smallest size compared to other datasets. This resizing process is formulated in Equations (1) and (2).

(1) $$h^{\prime }=565$$

(2) $$w^{\prime }=\frac{h}{565}\times w$$

where $h^{\prime }$ is the normalized height, $w^{\prime }$ denotes the normalized width, h represents the height, and w represent the width of the input image.

The next step was intensity normalization to overcome uneven lighting in the retinal image, which causes some areas other than the OD to appear brighter, thereby leading to localization errors. Intensity normalization is performed to minimize the effect of non-uniform lighting. Normalization was conducted by combining several morphological operations, as formulated in Equations (3)–(5). These operations were applied to the green layer of the image.

(3) $$I_{pre1}\left(I\right)=I+I_{bt}^{\theta }\left(I\right)+I_{bg}\left(I\right)$$

(4) $$I_{pre2}\left(I\right)=I_{op}^{\sigma }\left(I\right)+I_{bg}\left(I\right)$$

(5) $$I_{c}\left(I\right)=\frac{I_{pre1}\left(I\right)+I_{pre2}\left(I\right)}{2}$$

**Figure 3:**
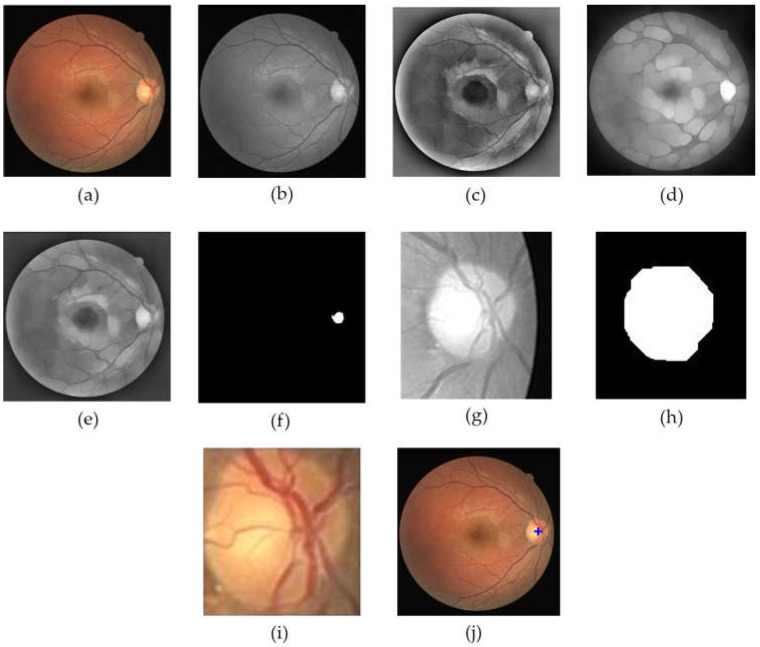
OD localization process, (**a**) input image, (**b**) grayscale image of *I* (**c**) *I_pre_*_1_ image, (**d**) *I_pre_*_2_ image, (**e**) $I_{c}$, (**f**) OD center area candidate (**g**) OD ROI (**h**) closing+opening result of binary OD ROI, (**i**) OD, (**j**) localization result of OD.

$I_{pre1}$ and $I_{pre2}$ represented precondition images used for uniform lighting while maintaining the brightness level of the OD area (Figure 3c,d). I represents the green layer of the resized input image. $I_{bt}^{\theta }\left(I\right)=\varepsilon^{\theta }\left(\delta^{\theta }\left(I\right)\right)-I$ was the morphological top-hat of *I*, performed with a disc structuring element, θ. Here, ε is morphological erosion and δ represents morphological dilation. *I_bg_* denotes the background image generated through filtering operations using an average filter. According to [30], the filter utilized was 89 × 89, in which $I_{pre2}$ was generated from the sum of $I_{op}$ and the background image, while $I_{op}^{\sigma }=\delta^{\sigma }\left(\varepsilon^{\sigma }\left(I\right)\right)$ was the morphological opening of *I* with structuring element of σ. The average of $I_{pre1}$ and $I_{pre2}$ produced an image called $I_{c}$ with more even lighting, and the optic disc area was maintained as shown in Figure 3e.

In order to obtain the OD ROI, *Ic* was first binarized through the thresholding operation on the $I_{c}$ image as conducted in [29], while the threshold value used was 0.89 of the maximum $I_{c}$ intensity. The threshold value was obtained through a series of empirically conducted experiments against a range of possible values. The resulting image is shown in Figure 3f. Then, the coordinates of this center point candidate were employed as the retinal image cropping center, while the crop size was $\frac{\left(w^{\prime }\times l^{\prime }\right)}{4}$. The retinal image cropping was performed on the red layer of the input image (Figure 3g). In the red layer, the OD was still clearly visible, and the presence of blood vessels did not have much effect [23].

The OD ROI was further processed to obtain the optic disc. The process began with contrast enhancement using Contrast-Limited Adaptive Equalization (CLAHE) and continued with opening morphology to remove blood vessels. The next step was binarization of the resultant image using the Otsu’s threshold. In order to obtain the candidate blob of the OD, morphological closing morphology followed by morphological opening were performed. Disc-structuring elements of radius 10 and 15 were used in these morphological operations (Figure 3h). Then, the resulting image was cropped according to the bounding box of the blob to obtain the OD (Figure 3i). The cropped optic disc image was called $I_{OD}$ and was used in the process of determining the temporal direction_._ Furthermore, the center point of the blob was determined to be the center point of the OD, which was then plotted on the retinal image to show the results of OD localization (Figure 3j). Figure 3 shows the series of results for the OD localization.

#### 2.2.2. Temporal Area Determination

The macula is a small area on the circular retina that has low intensity but does not contain blood vessels. According to [31], the center of the macula is located at 2.5 DD from the optic disc center. In [32], the retinal was vertically divided into temporal and nasal areas but horizontally divided into inferior and superior. One study found that the macular area is temporally located on the retina from the optic disc center [23]. Therefore, the temporal direction information was used in the proposed method to determine the macular ROI.

**Figure 4:**
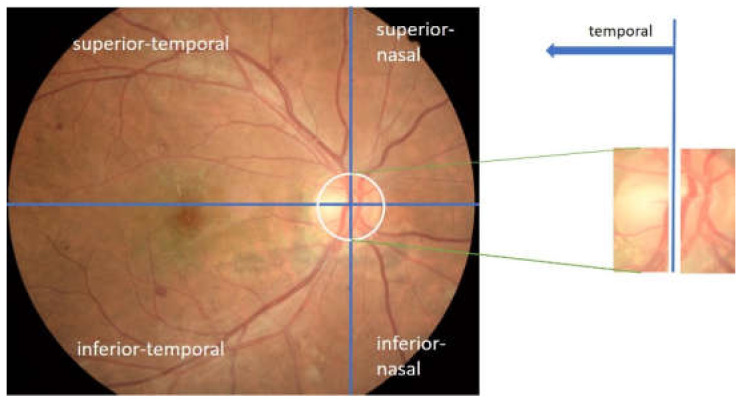
Temporal direction viewed from the appearance of blood vessels in the optic disc.

The main blood vessels of the retina converged at the optic disc. It was also observed that the blood vessels’ appearance has a unique character. For example, the blood vessels on the optic disc tended to gather at one side within the optic disc, indicating temporal and nasal directions on retinal images. This means that a relationship was formed between the nasal and temporal directions on the retinal image with the appearance of blood vessels. Moreover, when the optic disc was vertically divided by 2, the temporal direction was indicated by the area containing fewer blood vessels. The relationship between the temporal directions and the appearance of blood vessels on the retinal image viewed from the optic disc center is shown in Figure 4.

In this proposed method, the number of blood vessel pixels on the optic disc was utilized as a feature for determining the retinal image’s temporal direction. Furthermore, the optic disc area containing fewer blood vessels showed the temporal direction, and the pixel number was calculated on the binary image generated from the green layer of the OD ROI. This green layer was selected because it shows the blood vessels more clearly [23]. After the contrast enhancement process using CLAHE, a bottom-hat operation was conducted on the CLAHE image to further emphasize the blood vessels. A disc-structuring element of suitable pixel radius was used. This type of structuring element was appropriate for maintaining the shape of blood vessels. The size of the structuring element was selected to be slightly larger than the width of the blood vessel; 5 were selected.

**Figure 5:**
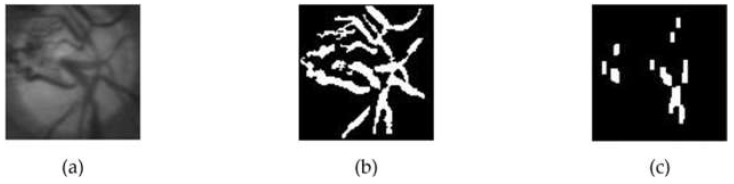
The extraction process of blood vessels on the optic disc; (**a**) optic disc on the green layer; (**b**) the extraction result of blood vessels on the optic disc; (**c**) the result of elimination of horizontal blood vessels.

It is important to note that blood vessel pixels were computed from black and white pixels generated through an adaptive thresholding process using Otsu. Unlike the technique employed by Zheng [23], this proposed method only involved vertically oriented blood vessels because there is a possibility that an optic disc is filled with blood vessels, thereby causing an error in detecting the temporal direction. On closer inspection, the blood vessels that tend to be vertical occupied the area opposite the temporal direction. Therefore, this method aims to eliminate horizontally oriented blood vessels before calculating the number of pixels in the optic disc, indicating that only the pixels showing the vertical blood vessels remain. The horizontal blood vessels were eliminated through a combination of opening and closing morphological operations. Therefore, a rectangular structuring element with a vertical orientation was used to maintain vertical vessels. Rectangular-shaped structuring elements of size 15 × 1 and 50 × 15, respectively, were used in this process. Figure 5 shows an example of a blood vessel extraction on the OD.

After the vertically oriented blood vessel images were obtained, the next step was determining the temporal area direction in the retinal image. The eliminated image was divided vertically into left and right segments, and then the number of white pixels on each segment was calculated. The temporal area was located in the segment with fewer blood vessels, indicating that when the left segment has more white pixels, the temporal direction was to the right, but when the right segment has more white pixels, the temporal direction was to the left.

#### 2.2.3. Macular ROI Determination

Macular ROI was determined to reduce the search area of the macular center and search time. In this method, the macular ROI was obtained through the following limits:

- The determination of macular ROI was based on the temporal direction. Furthermore, the macula located in the temporal area was obtained geometrically with reference to the OD center point [23].
- The macular center was 2.5 times of OD diameter from the OD center [5,20,33] and located slightly below the OD [34].

**Figure 6:**
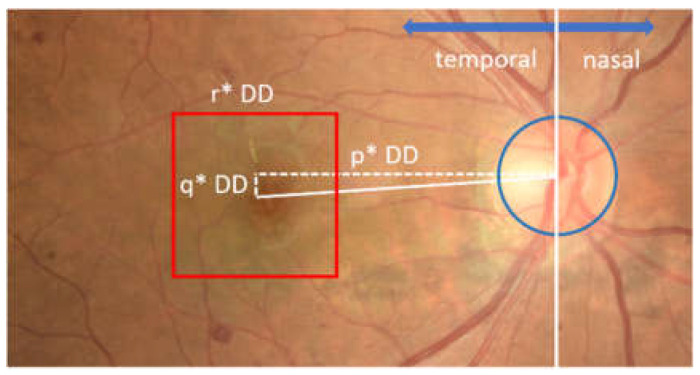
Illustration of macular ROI determination. The red box shows the macular ROI.

Using these limits, the central location of the macular ROI was determined in this study via Algorithm 1. Figure 6 shows an illustration of macular ROI determination. The inputs for Algorithm 1 were the temporal direction, center point of OD, and diameter of OD. The OD center had been obtained when performing OD localization, while the diameter of the OD was obtained by measuring the ratio between the width of the OD and the width of the retinal image. The experimental results showed that the appropriate OD diameter was $\frac{v^{\prime }}{12}$ where *v*′ was the width of the retinal in the image segmented by Otsu thresholding on the grayscale image. The use of these three parameters, as shown in Algorithm 1, could provide an optimal macular ROI.
**Algorithm 1:** Macular ROI determination**Input:** $direction_{temporal}$, *OD center coordinates (*$x_{OD},y_{OD})$*, OD diameter (DD)***Parameter:** *abscissa factor (p), ordinate factor (q), ROI box factor (r)*1: **if**  $arah_{temporal}=LEFT$ **then**2: $\;x_{M}\leftarrow x_{OD}-p\times DD$3: **else**4: $\;x_{M}\leftarrow x_{OD}+p\times DD$$5:\;y_{M}\leftarrow \;y_{OD}+q\times DD$6: dimROI← r×DD7: *determine the macular ROI with the center* ($x_{M},\;y_{M})\;and\;the\;size\;of$ dimROI

As shown in Figure 6, the ROI of the macula was determined in the form of a square shape with a size of *r* × DD, where *r* was the ROI box factor. In this study, the best values of the parameters *p*, *q*, and *r* were selected through parameter tuning, with possible values of *p* = {3.6, 3.8, 4.0}, *q* = {0,25, 0,5}, and *r* = {2.0, 2.25, 2.5}.

#### 2.2.4. Macular Center Extraction

After determining the macular ROI, its center was obtained through thresholding and morphological operations. The first step was to increase the contrast of the macular ROI image using CLAHE with a clip limit value of 1, while the value of parameter number of tile was [8 8]. In order to obtain the candidate blob of macula, binarization was performed on resultant. The threshold value was selected based on the maximum intensity, as formulated in Equation (6).

(6) $$\tau =0.98\times \max(I_{m})$$

**Figure 7:**
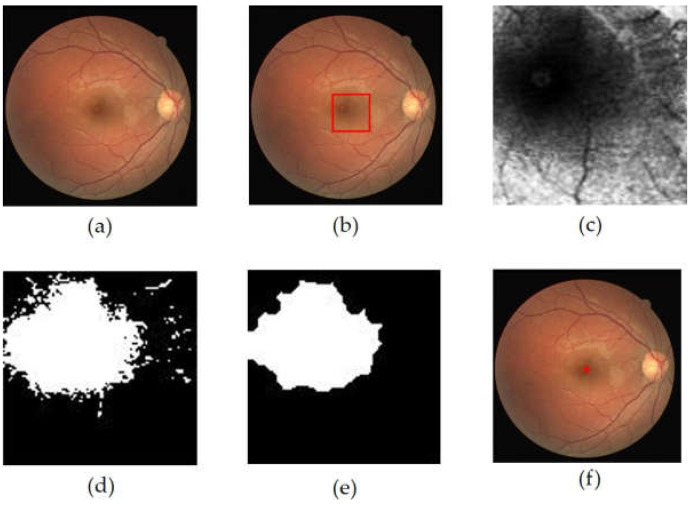
The extraction process results of the macular center point, (**a**) retinal image, (**b**) macular ROI localization result (red box), (**c**) macular ROI gray image, (**d**) macular ROI binarization result, (**e**) dilation + opening operation result, (**f**) macular center point detection result.

In order to minimize detection errors when the macula was in a dark area, a dilation morphological operation was performed. This operation was followed by an opening process, which enlarged the candidate of macular area. The largest candidate blob was selected as the macular area, and then the center of the blob was designated as the center of the macula. The extraction process results of the macular center point is shown in Figure 7.

## 3. Results

This method has been tested on the DRIVE, DiaretDB1, and Messidor public datasets, as well as the local dataset from JOGED.com. All images in the DiaretDB1, Messidor, and JOGED.com datasets were utilized in this test, but five images were not used for macular center detection in the DRIVE dataset. This is in line with [23], which stated that five images in the macular center area were invisible. The five images include image#4, image#15, image#23, image#31, and image#34. The macula center detection validation was conducted based on the Euclidean distance measure of the detected centers of macula from the ground truth (GT) centers. According to Medhi [35], if the automatically located center of macula was located less than of 50 pixels from ground truth, it was considered as a correct detection. The Euclidian distance formula is shown by Equation (7) [5,36]:

(7) $$distance=\sqrt{{\left(x_{system}-x_{GT}\right)}^{2}+{\left(y_{system}-y_{GT}\right)}^{2}}$$

**Figure 8:**
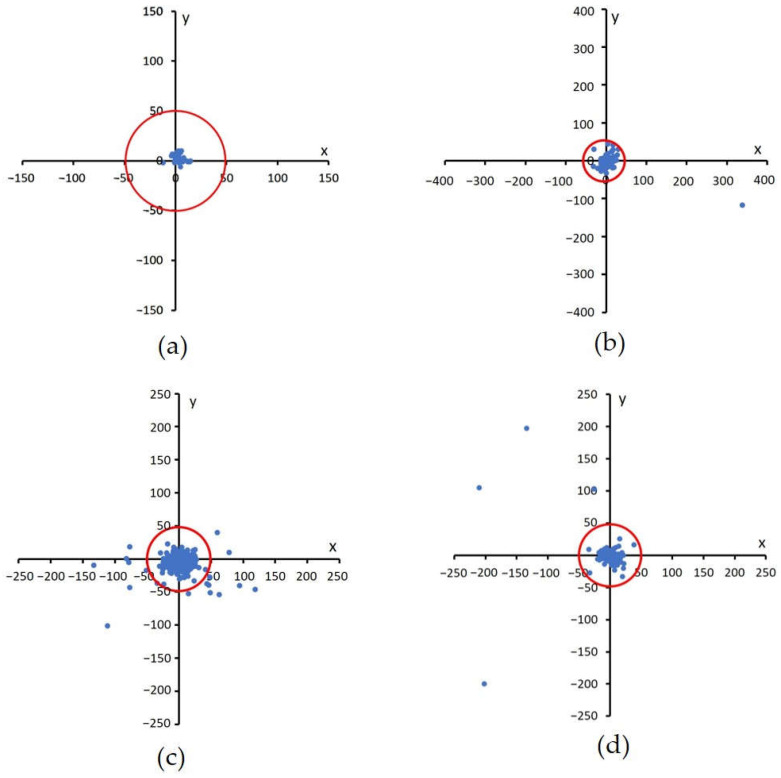
Euclidian distance distribution between the proposed method with GT. A red circle with a radius of 50 shows the acceptable distance limit for well-detected results, (**a**) DRIVE, (**b**) DiaretDB1, (**c**) Messidor, and (**d**) JOGED.com (accessed on 22 July 2022).

**Figure 9:**
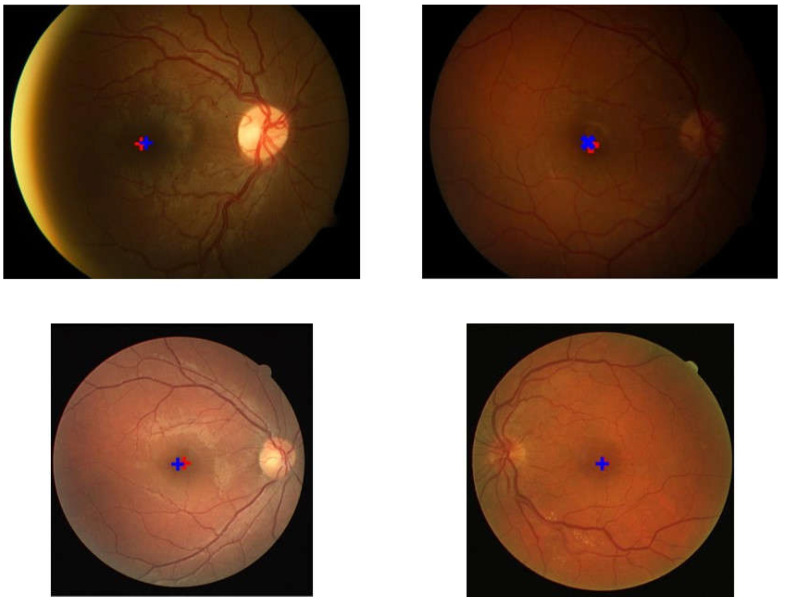
Examples of the detection results and location of the ground truth. The red ‘+’ symbol represents the detection result while the blue ‘+’ indicates the location of the ground truth.

These results were plotted on a Cartesian diagram, as shown in Figure 8. The ground truth value validated by experts was employed as the center point of the coordinates, while the points on the diagram showed the distribution of the points generated by the proposed method. In addition, a red circle with a radius of 50 indicated the acceptable distance limit for well-detected results. Therefore, the point distribution in this diagram showed the distribution of the accuracy of the proposed method in detecting the center of the macula in a dataset. There were several points that lie outside the red circle, as shown in Figure 8b–d. These points showed the coordinates of the macular center points that were not precisely detected. Examples of detection results are presented in Figure 9.

Based on experiments on the four datasets, this method works well and is stable. Out of the 35 images in the DRIVE dataset, the system was able to detect the macular center with 100% accuracy. Meanwhile, on the DiaretDB1 dataset, the proposed method achieved an accuracy of 98.87%, and on the Messidor dataset, 94.67%. Furthermore, the accuracy of this proposed method on the local JOGED.com dataset was 93%. The optimum value for the four datasets was successfully obtained by setting the abscissa distance to 3.8DD and the ordinate to 0.25DD. Consequently, the optimal macular ROI box size was obtained at 2DD × 2DD. The distance between the points recorded by the system and the ground truth produced the shortest average distance in the DRIVE with a value of 7.1, while those of DiaretDb1, Messidor, and JOGED.com were 15.8, 8.7, and 13.5, respectively.

The computation time test results showed that the method requires very little time. Specifically, it involved an average time of 0.34 s/image to detect the macular center in the DRIVE, while the DiaretDB, the Messidor, and the local JOGED.com were 0.57, 0.64, and 0.78 s/image, respectively. Table 1 shows that the time achieved by this proposed method exceeded others. This comparison was performed in studies that utilized the DRIVE, DiaretDB1, and Messidor datasets.

## 4. Discussion

The results showed that macular center detection by temporal area selection provided stable outcomes. It was observed that the selection of the right temporal direction provided a simple process for determining macular ROI. Furthermore, determining the optimal geometric macular ROI was able to provide stable detection results, which were not affected by image conditions that sometimes have uneven lighting.

In addition to the high-accuracy results, Table 1 also shows that the computational time of macular center detection in the proposed method surpassed others. For example, it significantly outperformed other methods on the DiaretDB1 dataset with large image size. Although the accuracy obtained for the DiaretDB1 and Messidor datasets was slightly below that of the other methods as reported in [24], the proposed method was much faster. Resizing and determining the macular ROI with an appropriate temporal reference are the keys to obtaining these results. This makes computing lighter and provides faster execution time, even with large images, namely the DiaretDB1 dataset.

The average computation time obtained by this method was slightly slower than the average time reported by Sedai, which had an average time of 0.4 s/image. However, the method in [23], which was based on deep learning, used high computational facilities supported by a 12 GB GPU. This can reduce computational time significantly, as stated by Chalakkal in [17].

**Figure 10:**
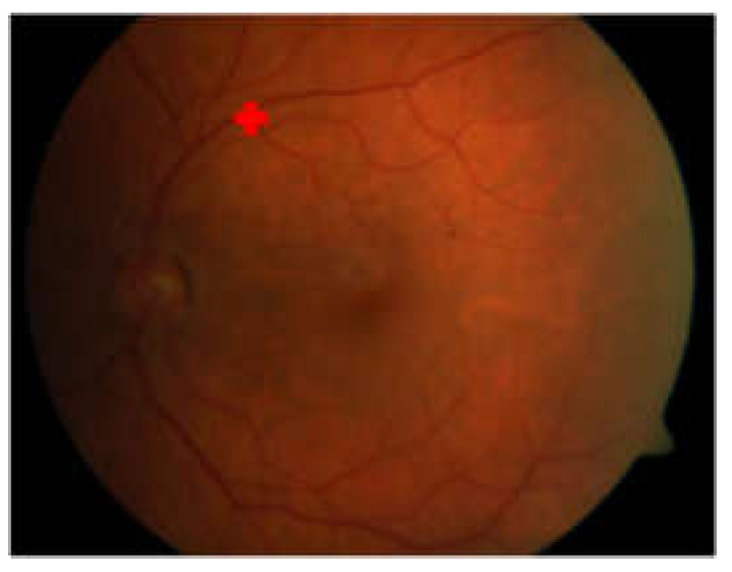
Macular center detection failure on image059.png of DiaretDB1.

The proposed method failed to detect the macular center of image059.png in the DiaretDb1 dataset and several images in the JOGED.com dataset. The detection failure of the macular center was caused by the inability to recognize the optic disc location due to irregular lighting in the image. It was observed that the non-optic disc area has a higher intensity compared to the optic disc. This condition causes OD candidate selection failures as well as OD localization and further leads to inaccuracy when determining the macular center. The geometric properties used in this method, which make the optic disc center a reference point, require precision in the localization of the optic disc. Therefore, failure in optic disc center localization resulted in failure when detecting the macular center. An example of a macular center detection failure that occured on image059.png in the DiaretDB1 dataset is shown in Figure 10.

## 5. Conclusions

This study described a new method for detecting the macular center location based on the temporal direction in retinal images. The proposed method was based on the geometric relationship between the macular area and the optic disc. The temporal direction determination provided optimal macular ROI, thereby leading to a detection process with low computation time and high accuracy. When compared to other methods, the results showed that the proposed method was faster while maintaining high accuracy.

## Figures and Tables

**Table 1:** Comparison of macular center detection results with other studies.

Method	DRIVE		DiaretDB1		Messidor		JOGED.com	
	Accuracy	Time (s)	Accuracy	Time (s)	Accuracy	Time (s)	Accuracy	Time (s)
Zheng [23]	100%	12	93.3%	12	-	-	-	-
Medhi [35]	100%	-	95.51%	-	97.98%	-	-	-
Chalakkal [17]	100%	25	95,5%	25	98.5%	25	-	-
Sedai [10]	100%	0.4	-	-	-	-	-	-
Romero-oraá [24]	100%	0.54	100%	14.55	99.67%	27.04	-	-
Proposed method	100%	0.34	98.78%	0.57	94.67%	0.64	93%	0.78

## Data Availability

Images from the public datasets, DRIVE, DiaretDB1 and Messidor were used in this article. The DRIVE dataset and documentation can be found at https://drive.grand-challenge.org/ (accessed on 15 April 2021), while DiaretDb1 can be found at https://www.it.lut.fi/project/imageret/diaretdb1/ (accessed on 21 June 2021). The Messidor dataset is kindly provided by the Messidor program partners at https://www.adcis.net/en/third-party/messidor/ (accessed on 29 October 2021). The ground truth of the macula center of both datasets is available at https://github.com/helmieaw/Ground-Truth-of-Macular-center (accessed on 22 September 2022). The JOGED.com local dataset was used in this article, but was not fully accessible.
